# Applying pytorch toolkit to plan optimization for circular cone based robotic radiotherapy

**DOI:** 10.1186/s13014-022-02045-y

**Published:** 2022-04-20

**Authors:** Bin Liang, Ran Wei, Jianghu Zhang, Yongbao Li, Tao Yang, Shouping Xu, Ke Zhang, Wenlong Xia, Bin Guo, Bo Liu, Fugen Zhou, Qiuwen Wu, Jianrong Dai

**Affiliations:** 1 Department of Radiation Oncology, National Cancer Center/National Clinical Research Center for Cancer/Cancer Hospital, Chinese Academy of Medical Sciences and Peking Union Medical College, Chaoyang Dist, 17 Panjianyuannanli Rd., Beijing, 100021 China; 2 Sun Yat-Sen University Cancer Center, State Key Laboratory of Oncology in South China, Collaborative Innovation Center for Cancer Medicine, Guangdong Key Laboratory of Nasopharyngeal Carcinoma Diagnosis and Therapy, Guangzhou, 510060 Guangdong China; 3 Department of Radiation Oncology, PLA General Hospital, Beijing, 100853 China; 4 Image Processing Center, Beihang University, Beijing, 100191 China; 5 Beijing Advanced Innovation Center for Biomedical Engineering, Beihang University, Beijing, 100083 China; 6 Division of Radiation Physics, Department of Radiation Oncology, Duke University Medical Center, Box 3295, Durham, NC 27710 USA

**Keywords:** Automatic differentiation, Lasso, Group lasso, Plan optimization, CyberKnife

## Abstract

**Background:**

Robotic linac is ideally suited to deliver hypo-fractionated radiotherapy due to its compact head and flexible positioning. The non-coplanar treatment space improves the delivery versatility but the complexity also leads to prolonged optimization and treatment time.

**Methods:**

In this study, we attempted to use the deep learning (pytorch) framework for the plan optimization of circular cone based robotic radiotherapy. The optimization problem was topologized into a simple feedforward neural network, thus the treatment plan optimization was transformed into network training. With this transformation, the pytorch toolkit with high-efficiency automatic differentiation (AD) for gradient calculation was used as the optimization solver. To improve the treatment efficiency, plans with fewer nodes and beams were sought. The least absolute shrinkage and selection operator (*lasso*) and the *group lasso* were employed to address the “sparsity” issue.

**Results:**

The AD-S (AD sparse) approach was validated on 6 brain and 6 liver cancer cases and the results were compared with the commercial MultiPlan (MLP) system. It was found that the AD-S plans achieved rapid dose fall-off and satisfactory sparing of organs at risk (OARs). Treatment efficiency was improved by the reduction in the number of nodes (28%) and beams (18%), and monitor unit (MU, 24%), respectively. The computational time was shortened to 47.3 s on average.

**Conclusions:**

In summary, this first attempt of applying deep learning framework to the robotic radiotherapy plan optimization is promising and has the potential to be used clinically.

**Supplementary Information:**

The online version contains supplementary material available at 10.1186/s13014-022-02045-y.

## Introduction

Robotic radiotherapy (CyberKnife, Accuray Inc., Sunnyvale, CA) enlarges treatment space and improves delivery versatility upon the standard linear accelerator, making it ideal for the delivery of stereotactic radiosurgery (SRS) [1, 2] and stereotactic body radiotherapy (SBRT) [3, 4]. The compact linac head is mounted on a highly accurate and reliable robotic arm. The robotic arm travels along the path connecting a set of pre-programmed discrete points (referred as node) distributed non-coplanarly. By orientating the linac head at these nodes, any beam pointing to the target could be delivered. The original collimator system included 12 diameter fixed circular cones, which was later upgraded to the variable aperture IRIS collimator [5]. Recently the vendor introduced multi-leaf collimator (MLC) to the system. Although the MLC increased the flexibility for field size and improved the treatment efficiency for irregular shaped targets, the circular cones are still in use widely due to its highly conformal dose distribution [6]. Furthermore, the MLC system has not yet been widely adopted, with only about 20% systems installed globally at the current time according to the vendor. Therefore, any improvement in the circular cone based plan optimization will have a big impact in the clinical operation of these systems, which is the aim of this study.

The non-coplanar treatment space improves the delivery versatility but also leads to prolonged treatment time. A typical treatment plan may use up to 100 beams/nodes and 2–3 size cones to get a conformal dose distribution, and the treatment time may take 30 min to 1 h, depending on the tumor sites [7]. The most effective approach to shorten treatment time is to use fewer beams, nodes and cones in the plan. This is because the beam positioning (including the switch of cones, the reorientation within each node and the robotic movement between the nodes) time, rather than the beam-on time takes the major part of the treatment duration. The need to improve the treatment efficiency means to reduce the number of beams and nodes without jeopardizing the quality of plan. Previously, we presented a singular value decomposition accelerated linear programming (SVDLP) optimization method [8]. This method used the least absolute shrinkage and selection operator (*lasso*) [9] for beam reduction. The number of nodes may also be reduced with the decrease of beams, but the issue was not fully addressed, at least not directly. Naturally, the beams delivered at the same node belong to the same “group”. The aim is to search for a sparse solution, for both the individual (beam) and group (node) aspects. In this study, we adopted both the *lasso* and the *group lasso* [10] regularization terms to address the “sparsity” issue.

Gradient based optimization approaches methods were commonly used for planning optimization [11, 12]. The computation of derivatives is often the most time-consuming step. Based on the chain rule of differential calculus, automatic differentiation (AD) replaces the domain of variables with the computation graph, and had been used for intensity-modulated radiation therapy (IMRT) planning optimization [13] as early as 2006*.* But back then, the limited packages and supported programming language hindered the application [14]. Recently AD received increasing attention with the emergence of deep learning (DL), as it had been used for the training of large-scale network. The recent development of DL also provided several well-established framework such as tensorflow [15], caffe [16] and pytorch [17], which saved the trouble of “reinventing the wheel” for regular investigators. In this study, we adopted pytorch framework for the circular cone based planning optimization, and explained how to use the well-validated DL framework for inverse planning.

The presented optimization approach used AD to compute derivatives, and the *lasso* and the *group lasso* to address the “sparsity” issue. Therefore it is later referred as AD-S approach for simplicity. Twelve (12) clinical cases of different complexities (brain and liver) were used to validate the AD-S approach. Currently, the commercial treatment planning system (TPS, MultiPlan) implements a sequential optimizer (SO) [18, 19]. In November 2018, the vendor released a new TPS (Precision v2.0), and upgraded the optimizer to VOLO. VOLO implemented graphical processing unit (GPU)-based dose calculation, replaced the sequential optimization with a single cost function integrating all clinical goals (widely used in the field of IMRT/VMAT optimization), and pruned the beams with lower monitor unit (MU) during optimization. As far as the authors know no centers or institutions in China has the Precision 2.0 installed currently. Therefore a direct comparison with Precision is not possible, and indirect comparison based on published data were performed and discussed.

## Methods and materials

In this section, the AD-S approach and optimization experiments were presented. First the procedure of beam initialization was introduced briefly. Then the optimization model and the *lasso* and *group lasso* regulation terms were described in details. The topology and transformation of the optimization model was presented in the following sub-section. Finally the patient data implementation details were described.

### Beam initialization

Generally, two methods are used for beam initialization. The first one is to start with a sub-set of randomly initialized candidate beams, then eliminate the beams with lower weights and add new candidate beams after each iteration of the optimization. The SO of Multiplan system used this “drop-and-pick” mechanism. Due to the intrinsic heuristic nature, it is inefficient to cover the complete treatment space. The other method is to initialize candidate beams to cover the entire beam space, and use all initialized beams for optimization simultaneously. This method takes full advantage of the treatment space but also means greater computational cost. The second scenario was adopted in our previous work [8] with a novel technique for beam initialization. The mechanism simulates the process of moving the equivalent dose taper to cover the entire target volume. The candidate beams were initialized with the center at the edge of adjacent beams. Both dosimetric analysis and patient data-based experiments proved that the mechanism could thoroughly cover the beam space without introducing redundancy. The same mechanism was adopted in this study.

### Optimization model

With the initialized candidate beams, the dose of each voxel (*D*_*i*_) is expressed as:

1 $$D_{i} = \sum\limits_{j = 1}^{n} {d_{ij} w_{j} } ,\quad i \in \{ 1,2, \ldots ,m\} ,\;w_{j} \ge 0$$

2 $$W = (w_{1} ,w_{2} , \ldots ,w_{n} )^{T}$$

*m* and *n* is the number of voxels and candidate beams, respectively. *d*_*ij*_ is the dose delivered to *i*th voxel by the *j*th beam, *w*_*j*_ is the weight of the *j*th beam. *W* is the weight vector. For each optimization objective, the cost function is of the general format:

3 $$f = \frac{1}{N}\sum\limits_{i} {(D_{i} - D_{i}^{0} )^{2} \times g(D_{i} ,D_{i}^{0} )}$$

*N* is the number of voxels of certain region of interest (ROI), *g* is the condition function varying with the objectives. $D_{i}^{0}$ is the prescription dose for planning target volume (PTV) or the tolerance dose for organs at risk (OARs). Table 1 lists the condition functions of some typical objectives.

**Table 1:** Condition functions of typical objectives

Objectives	*g*
Max dose	$H(D_{i} - D_{i}^{0} )$
Min dose	$H(D_{0} - D_{i}^{0} )$
Uniform dose	1
Max DV	$$\left\{ {\begin{array}{*{20}c} {H(D_{i} - D_{i}^{0} )} & {{\text{head}}} \\ 0 & {\text{others }} \\ \end{array} } \right.$$
Min DV	$$\left\{ {\begin{array}{*{20}c} {H(D_{i}^{0} - D_{i} )} & {{\text{tail}}} \\ 0 & {{\text{others}}} \\ \end{array} } \right.$$

*H* is the Heaviside function. For dose volume (DV) objectives, head and tail refer to the voxels at the top and end position sorted by dose in descending order

The total objective function is the summation of all objectives and the *lasso* and *group lasso* regularization terms:

4 $$F = \alpha_{k} \sum\limits_{k} {f_{k} } + \lambda_{1} \left| W \right| + \lambda_{2} \sum\limits_{l} {\left\| {W_{l} } \right\|_{2} }$$

5 $$\left| W \right| = \sum\limits_{j} {\left| {w_{j} } \right|} ,\;\left\| {W_{l} } \right\|_{2} = \sqrt {\sum\limits_{j \in l} {w_{j}^{2} } }$$

where *α* and λ are the weight of objective function and regularization terms, respectively. | | and $\left\| {\,} \right\|_{2}$ represents the operation of L1 and L2 norm, respectively. *W*_*l*_ is the weight vector composed by the weights of beams belonging to the *l*th node.

### Topologizing optimization model and training practice

**Fig. 1:**
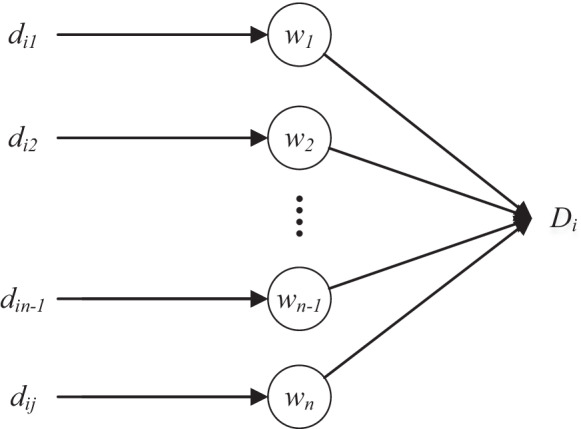
Network of dose calculation (Eq. 1)

The total dose (*D*_*i*_, *i* ∈ (1,2,…, *m*)) of each ROI voxel is contributed by all beams (*d*_*ij*_, *j* ∈ (1,2,…,*n*)), which is calculated as the weighted (*w*_*j*_) linear combination of *d*_*ij*_ (Eq. 1). This operation can be topologized into a simple feed-forward neural network, as shown in Fig. 1. This network is of the simplest architecture: it contains no hidden layers, and only the basic input and output layers. The neuron weight represents the beam weight. In this way, the optimization of beam weight is transformed into the tuning of neuron weight, i.e. the treatment planning optimization is converted into “network training”.

As listed in Table 2, the “training” is different from typical DL network: (1) For typical DL, the network was trained and tested on training and testing datasets, respectively. For treatment planning, the network was trained for each single patient. Once the training was completed, the optimization was also finished. No testing process was required. (2) For DL network training, only a small subset of training dataset was used (mini-batch strategy) for each iteration. But for treatment planning network, the dose delivered to all voxels by all beamlets (*d*_*ij*_, *i* ∈ {1, 2, …, *m*}, *j* ∈ {1, 2, …, *n*}) were feed into the network to calculate the loss function for each iteration. (3) For DL, the desired results were the whole network. In other words, the goal was to obtain correct results via the network; the exact weight of each neuron was not the concern. But for this work, the weight of each neuron was the weight of corresponding beam, which were the desired results.

**Table 2:** Comparison of the training of treatment planning and typical DL networks

	Typical DL	Treatment planning
Train and test process	Train and test on different datasets, separately	Train (optimize) for each patient without testing process
Input data of training iteration	Mini-batch of training dataset	All data of one patient
Desired results	Network	Neuron weight

With this transformation, the well-established framework for DL could be tapped to solve the treatment plan optimization problem. The regularization terms are designed to filter out the beams of very limited contribution to the plan. In practice, it was found that the weights of these beams were reduced to a very small value but not exactly zero. In order to reduce the computational cost, these beams are removed during optimization, i.e. the network is dynamically adjusted by trimming the neurons with marginal weights. The threshold of trimming beam weight was set to 0.01 MU. In addition, an early stopping criterion was adopted: the iteration is terminated when the number of beams does not decrease within certain consecutive iterations. The number of consecutive iterations is set to 10.

### Materials: patient data and machine parameters

The Cyberknife system provides two sets of pre-defined nodes for head and body treatment. The treatment position determined which set of nodes to use. The AD-S approach was validated on two types of cases: 6 brain and 6 liver cancer cases. For the brain cases: patient 1 was lymphoma case, patient 3 and 5 were glioma cases with large PTV, and the rest were metastases. For each case, the AD-S plan used the same set of nodes as MLP plan. Table 3 listed the patient data, including the prescription dose (*D*_*P*_), fraction dose, number of fractions, volume of PTV (V_PTV_), number of voxels of all ROIs, the sizes of used cones, the number of available nodes and initialized beams. Because the computational time of AD-S approach is directly related to the planning parameters, it was also listed. The spacing of optimization dose grid was 2 mm × 2 mm × 2 mm, and the ROI was sampled to 2 mm, correspondingly. The MLP plans were designed and approved by experienced physicists and physicians. The AD-S plans were optimized using MLP plans as reference. The optimization terminated if the obtained plans were no inferior to MLP plans. The size of cones were selected by experienced planner according to the size and shape of PTV. The AD-S approach used the same set of cones with the MLP plans.

**Table 3:** Summary of patient data

	*Dp* (cGy)	Fraction dose (cGy)	Fraction	V_PTV_ (cc)	No. of voxels	Cone (mm)	No. of nodes	No. of beams	Time (S)			
									Ini	Cal	Opt	Total
Brain	2000	500	4	6.9	21,901	7.5/10	121	7091	2.3	10.6	57.4	70.3
	2000	500	4	17.6	17,418	12.5/25	71	2417	2.5	3.7	17.7	23.9
	2000	400	5	47.3	28,104	12.5/20	121	7907	2.7	12.5	69.6	84.8
	2000	500	4	25.9	18,923	12.5/35	79	3676	1.9	4.4	36.9	43.3
	3000	500	6	53.6	27,699	25/35	87	1281	0.3	2.0	18.8	21.1
	3000	600	5	14.8	19,109	12.5/25	122	3173	2.9	4.7	36.9	44.5
Liver	5000	500	10	50.3	40,332	20/30	80	1807	6.9	3.5	28.4	38.8
	5000	500	10	15.9	38,992	15/25	78	1408	2.0	2.5	24.7	29.1
	5500	550	10	38.6	32,250	20/25	80	1893	5.4	3.2	23.5	32.1
	5000	500	10	18.7	43,506	15/25	79	1591	2.5	2.8	29.0	34.3
	5000	500	10	125.6	52,144	20/30	94	4093	3.3	7.5	81.5	92.4
	5000	500	10	168.4	56,215	30/40	80	1764	3.4	3.8	45.4	52.5

The time column lists the time of candidate beam initialization (Ini.), dose calculation (Cal.), optimization (Opt.) and the total time

The beam initialization was implemented in C++, and dose was calculated using ray-tracing method on GPU. Plans evaluation was performed on an in-house platform, which was developed and validated in our previous study [8, 20]. The MLP plans were also imported to the platform, and all the following mentioned plan evaluation indices were calculated under the same resolution (2 mm × 2 mm × 2 mm) with AD-S plans. Please note: since the resolution was different, it may result in slightly changes in these indices. The optimization was implemented with pytorch (v0.4.0, Paszke et al. [17]). The critical codes could be found in the suppelmental material. The example of using pytorch for treatment planning optimization was illustrated with a simple case in Additional file 1. Among the available optimizers in pytorch, it was found that the L-BFGS is the fastest algorithm with satisfactory robustness. Therefore the L-BFGS optimizer was selected in this study. Note that: because the L-BFGS includes certain logical operations and the network is simple and shallow, it executes faster on CPU than on GPU. So the optimization was performed on CPU in this study. All computations were performed on a desktop computer with Intel Xeon E5-2620 processor and NVIDA GeForce GTX 1080ti GPU.

## Results

In this section, the detailed result of one sample liver case, including the objective value and the numbers of nodes and beams during optimization, was first presented. And then the evaluation and comparison of the treatment plans generated by the AD-S approach (later referred as AD-S plans) and MLP system (later referred as MLP plans) on the cohort of patients were presented.

### Liver case

**Fig. 2:**
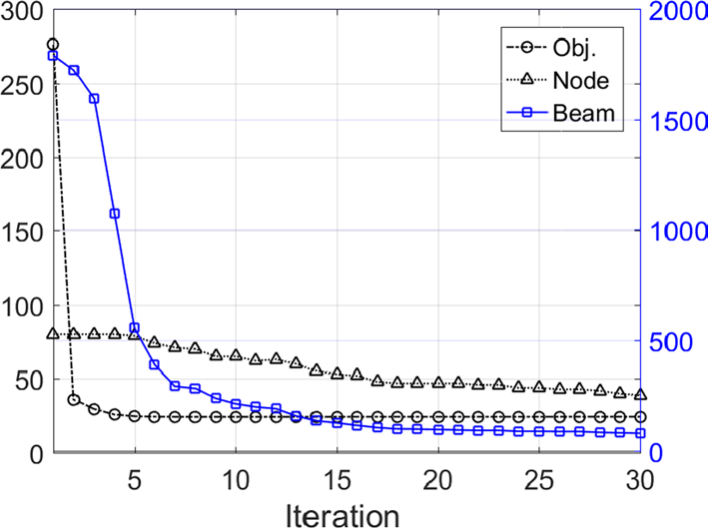
Optimization results of the first liver case. The vertical axis shows the loss value and the number of nodes in black on the left side, and the number of beams in blue on the right side. The lines were plotted in corresponding color. The final loss value, number of nodes and beams were also annotated in the legends

Figure 2 showed the objective value (*F* in Eq. 4) and the numbers of nodes and beams as functions of iterations of the first liver case. The objective dropped dramatically at the first iteration, but much slower for the subsequent iterations. The number of beams decreased rapidly at the first several iterations, and continued to decrease, and remained the same for the last 10 iterations due to the early stop strategy. The number of nodes decreased gradually through the whole optimization. The beam and node reduction does not increase the objective value, indicating the number of beams and nodes was reduced without jeopardizing the plan quality.

### Evaluation and comparison

Table 4 compares the evaluation indices between AD-S and MLP plans. For the brain cases, the AD-S plans reduced the number of nodes, beams and MU by 26%, 17.5% and 26% on average. For liver cases, these indices were reduced by 29%, 18% and 22.2%. And for all cases, the reduce ratio was 27%, 18% and 24%. The conformity index (CI) is defined as the ratio of the volume covered by *D*_*p*_ over the volume of PTV (V_PTV_). For the brain, liver and all cases, CI was improved by 18%, 19% and 19%. The homogeneity index (HI) is defined as the ratio of the maximum dose (*D*_*max*_) within PTV over *D*_*p*_. On average, the HI of AD-S plans for brain cases was slightly lower than MLP plans but slightly higher for liver cases. Paired *t*-test was performed on the indices of all AD-S plans against MLP plans. The results indicated that the reduction on the number of nodes and beams, MU and CI was significant. The change of HI was not significant. We would like to point out that: only a soft constraint was imposed on *D*_*max*_, and no certain height (like 150% of *D*_*p*_) was compulsory for either MLP or AD-S plans. We only limited the maximum dose of PTV at 110% of prescription dose with a relative low weight. The average computational time of AD-S approach was 39.6 s (23.0–56.1 s) for brain cases, 38.8 s (20.8–56.7 s) for liver cases and 39.2 s (27.5–56.7 s) for all cases.

**Table 4:** Number of nodes, beams, MU, CI, D_max_ and HI comparison of AD-S and MLP plans

	No. of nodes			No. of beams			MU			CI			HI		
	AD-S	MLP	Ratio	AD-S	MLP	Ratio	AD-S	MLP	Ratio	AD-S	MLP	Ratio	AD-S	MLP	Ratio
Brain	52	72	0.72	83	123	0.68	26,759.4	40,348.0	0.66	1.06	1.28	0.83	1.16	1.11	1.05
	55	73	0.75	151	179	0.84	23,905.8	28,069.0	0.85	1.09	1.34	0.81	1.25	1.34	0.93
	63	80	0.79	119	146	0.82	24,242.7	34,516.5	0.70	1.08	1.39	0.78	1.21	1.29	0.94
	59	75	0.79	130	141	0.92	21,111.5	27,985.2	0.75	1.12	1.26	0.89	1.11	1.10	1.01
	54	75	0.72	134	151	0.89	14,099.2	18,766.1	0.75	1.15	1.43	0.80	1.19	1.21	0.98
	65	98	0.66	160	199	0.80	26,676.5	36,531.6	0.73	1.08	1.30	0.83	1.17	1.18	0.99
	0.74 (0.70–0.78)			0.83 (0.76–0.89)			0.74 (0.69–0.79)			0.82 (0.79–0.85)			0.98 (0.95–1.02)		
Liver	39	51	0.77	80	100	0.80	37,693.0	46,693.2	0.81	1.10	1.29	0.85	1.19	1.17	1.02
	46	65	0.71	118	135	0.87	44,690.9	48,487.5	0.92	1.09	1.35	0.81	1.09	1.09	1.00
	25	38	0.66	81	100	0.81	36,108.4	46,468.6	0.78	1.07	1.32	0.81	1.22	1.20	1.02
	37	59	0.63	116	143	0.81	61,853.9	76,896.0	0.80	1.07	1.4	0.76	1.11	1.09	1.02
	48	62	0.77	114	130	0.88	50,577.6	82,111.8	0.62	1.13	1.38	0.82	1.27	1.31	0.97
	39	53	0.74	72	94	0.77	40,155.7	54,089.8	0.74	1.05	1.35	0.78	1.44	1.41	1.02
	0.71 (0.66–0.76)			0.82 (0.79–0.86)			0.78 (0.70–0.86)			0.81 (0.78–0.83)			1.01 (0.99–1.02)		
All	0.73 (0.70–0.76) *p*-value: 2.42 × 10^–7^			0.82 (0.79–0.86) *p*-value: 1.91 × 10^–6^			0.76 (0.71–0.81) *p*-value: 3.25 × 10^–4^			0.82 (0.80–0.83) *p*-value: 4.70 × 10^–9^			1.00 (0.98–1.02) *p*-value: 0.56		

The ratio column was the ratio of the AD-S over MLP. The average ratio along with 95% confidential interval (CI) was listed at the bottom of each cases

**Fig. 3:**
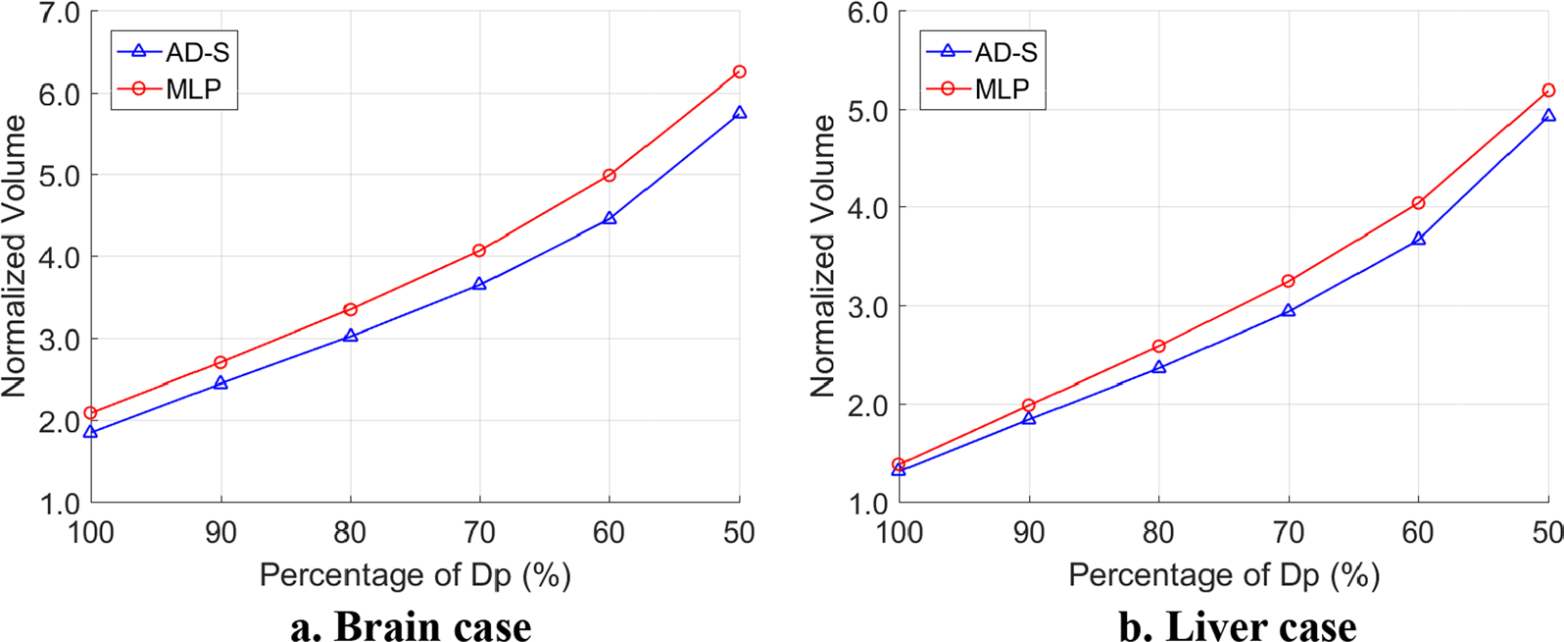
Dose gradient comparison. The horizontal axis is the relative dose normalized to *D*_*p*_, and the vertical axis is the volume covered by corresponding dose normalized to V_PTV_.

Figure 3 showed dose gradient comparison of AD-S and MLP plans. For each case, the volume covered by 50–100% of *D*_*P*_ (V_50_–V_100_) was normalized to corresponding V_PTV_. As shown in Fig. 3, the AD-S curve was under the MLP curve for each type of case, indicating that the AD-S plans achieved higher/better dose gradient around PTV, i.e., more rapid dose fall-off.

Tables 5 and 6 listed the maximum and average dose of the OARs of the brain and liver cases, respectively. Table 5 also listed the V_5_, V_10_ and V_20_ of the liver organ. The dose of AD-S plan greater (worse) than the MLP plan was denoted in bold. For the brain cases, the dose of AD-S plans was greater for 8 out of 60 items. Out of the 8 items, 5 had deviations less than 30 cGy, and the maximum deviation was 166 cGy. For the liver cases, the dose of AD-S plans was greater for 14 out of 96 items. Out of the 14 items, 11 had deviations less than 70 cGy, and the maximum deviation was 191.9 cGy. These demonstrated that the AD-S plans had better overall OAR sparing.

**Table 5:** OAR protection of the brain cases

Patient			1	2	3	4	5	6
Brain-PTV	D_max_	AD-S	1091.9	2086.7	**960.6**	1550.6	**3384.7**	3095.0
		MLP	1157.6	2242.8	855.0	1698.2	3356.7	3086.5
	D_mean_	AD-S	132.6	180.9	162.2	180.9	621.2	327.9
		MLP	150.3	204.0	189.6	255.2	664.4	404.7
Brain stem	D_max_	AD-S	487.0	1603.9	636.1	1613.2	892.6	1167.2
		MLP	573.4	1698.7	705.2	1623.8	914.4	1272.3
	D_mean_	AD-S	107.1	424.9	235.7	416.5	325.8	348.6
		MLP	149.3	675.2	331.1	674.3	486.2	635.4
Left optic nerve	D_max_	AD-S	469.5	72.9	772.6	1046.4	67.6	42.4
		MLP	844.8	581.8	871.8	1590.7	469.0	76.7
	D_mean_	AD-S	149.7	**155.0**	383.0	273.4	31.3	**38.3**
		MLP	297.6	135.0	610.5	407.4	144.4	22.2
Right optic nerve	D_max_	AD-S	2170.6	**1789.9**	920.2	320.8	71.1	42.4
		MLP	2173.4	1671.8	1046.4	1092.6	139.4	76.7
	D_mean_	AD-S	1505.2	**598.0**	439.1	71.2	35.6	**38.3**
		MLP	1542.5	432.0	565.5	239.6	50.7	22.2
Optic chiasm	D_max_	AD-S	2089.9	**1312.1**	860.6	2014.6	85.3	42.4
		MLP	2136.5	1310.8	1006.7	2114.2	471.6	216.7
	D_mean_	AD-S	1277.4	851.7	389.5	1637.0	85.3	40.3
		MLP	1391.0	889.5	573.5	1889.1	471.6	67.0

The unit of dose is cGy

**Table 6:** OAR protection of the liver cases

Patient			1	2	3	4	5	6
Liver-PTV	V_5_	AD-S	53.6	45.5	39.2	31.0	58.7	74.5
		MLP	63.1	58.9	44.2	39.8	69.2	91.7
	V_10_	AD-S	35.7	23.2	18.3	12.2	33.3	57.9
		MLP	39.1	25.5	19.7	13.8	45.5	65.6
	V_20_	AD-S	16.8	8.0	7.1	3.7	6.9	29.1
		MLP	18.1	8.5	7.2	4.0	16.7	30.8
	D_mean_	AD-S	1150.9	740.4	662.8	523.8	891.1	1539.5
		MLP	1210.6	845.4	724.7	557.6	1206.5	1768.4
Cord	D_max_	AD-S	45.8	155.2	410.2	896.1	1054.1	512.6
		MLP	57.5	240.4	500.5	1150.2	1168.1	836.7
	D_mean_	AD-S	**208.3**	71.9	155.2	170.6	432.1	273.2
		MLP	148.6	71.9	201.0	220.6	657.2	387.9
Stomach	D_max_	AD-S	272.2	739.1	671.1	242.4	**2747.3**	1133.4
		MLP	669.0	1045.5	989.5	387.8	2586.9	1309.8
	D_mean_	AD-S	157.7	164.8	207.4	314.5	1155.2	316.4
		MLP	200.8	246.4	281.9	406.0	1229.1	387.9
Bowel	D_max_	ADS	785.6	671.6	262.0	196.2	1530.3	461.8
		MLP	831.5	773.8	558.9	940.5	1671.8	925.2
	D_mean_	ADS	58.5	**81.1**	92.5	**96.0**	366.8	233.6
		MLP	63.5	47.7	122.9	60.9	450.9	236.8
Duodenum	D_max_	ADS	**208.4**	874.7	1133.8	**98.0**	**2739.7**	1335.0
		MLP	198.9	1165.8	1237.2	57.5	2547.8	1438.6
	D_mean_	ADS	65.4	155.9	462.2	168.4	**1059.2**	425.7
		MLP	68.6	210.0	586.0	219.5	1057.1	523.6
Esophagus	D_max_	ADS	742.3	**301.9**	214.1	849.3	1551.5	**640.4**
		MLP	858.7	294.9	329.3	1089.8	2176.8	619.2
	D_mean_	ADS	280.1	105.4	124.4	232.2	534.0	405.2
		MLP	343.1	131.1	163.6	395.8	722.2	420.7
Right kidney	D_max_	ADS	278.9	301.8	592.0	**634.2**	1263.4	1312.8
		MLP	549.5	321.4	616.8	471.7	1737.2	1480.6
	D_mean_	ADS	**161.2**	**125.0**	285.8	**121.2**	395.6	387.5
		MLP	114.9	59.6	317.9	83.1	524.8	771.7

The unit of dose is cGy, and the unit of V_x_ is %

**Fig. 4:**
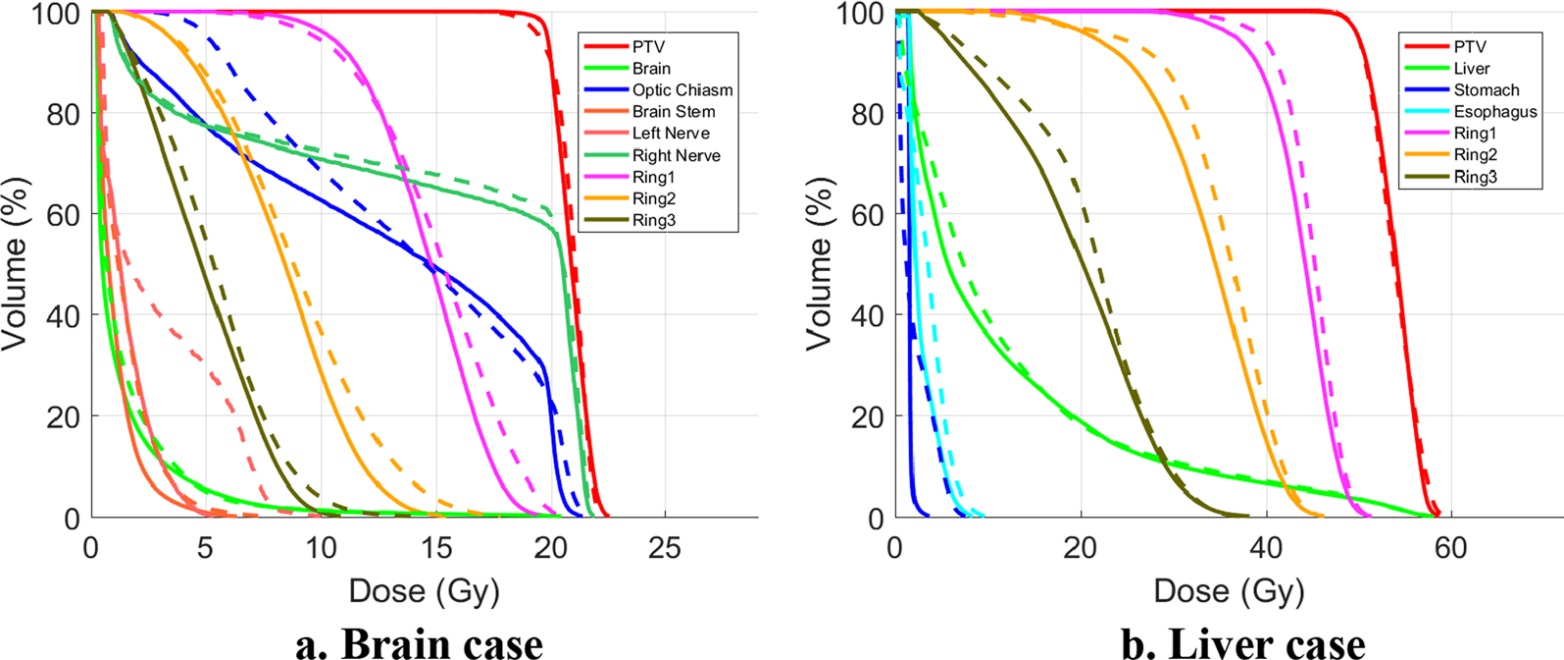
DVHs of the first liver and brain cases. The AD-S plans were plotted in solid lines and MLP plans in dash lines

**Fig. 5:**
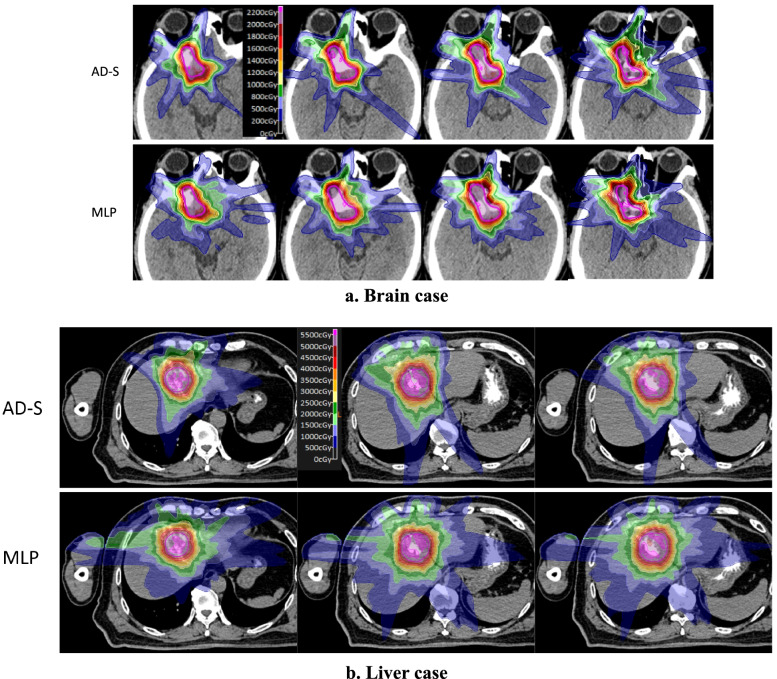
Dose distribution of the first brain and liver cases. PTV was depicted in purple solid thick line, and the isodose line in thin lines with different colors.

Figures 4 and 5 showed the dose volume histograms (DVHs) and the dose distribution of the first brain and liver cases, respectively. For the brain case, the minimum dose with PTV of MLP plan was significantly lower, corresponding with the “round-ended” the DVH curve. This may be caused by the node or beam reduction procedure of MLP, which was to remove the nodes or beams with lower weight after weight optimization. For the ADS approach, the beam and node reduction was implemented during optimization, which would not jeopardize the plan quality. As shown in Fig. 4, the dose of the rings outside PTV of the ADS plans was lower than the MLP plans, indicating more rapid dose fall-off, which could also be observed on the dose distribution comparison of Fig. 5.

## Discussion

In this study, we topologized the optimization problem into a simple feed-forward neural network, thus converted the treatment plan optimization into network training, and furthermore utilized the automatic differentiation approach for network training. In this way, the well-established pytorch DL framework was used. Our results demonstrated that the framework could handle both the brain and liver cases with different complexities. In addition, the optimization speed was also satisfactory. As far as we know, this was the first attempt to successfully adapt machine learning toolkit to the cone-based treatment plan optimization.

Using pytorch for inverse planning is naturally different from the typical application of DL. In standard DL, the weight of single neuron has no clear meaning, and the whole network acts like a “black box”. For this application, the weight of each neuron represents the weight of each candidate beam. The other difference is the practice of “network training”. For DL, over-fitting may be the most challenging issue, meaning that the network may have excellent performance on the training dataset, but poor performance on testing dataset. The mini-batch training strategy is used to avoid over-fitting, which is to randomly select very few samples for training at each iteration. In this study, the beam weights were optimized separately for each plan. This means the network was trained from scratch, and all voxels were training dataset, thereby avoiding the issue of over-fitting. Therefore the full-batch training strategy was used in our strategy.

For results comparison, since the authors do not have access to the latest VOLO, we compared AD-S with the MLP system. The reduction rate of the AD-S approach over MLP was 27.5% (MU), 17.6% (node) and 24% (beam), which was comparable with VOLO according to published studies. Zeverino et al. reported the reduction rate of VOLO over MLP was 36% (MU), 14% (node) and 31% (beam) [21]. Schüler et al. found that the MU and beam reduction rate was 21.8% and 22.0% for 6D skull tracking plans, and 28.1% and 28.4% for Xsight spine tracking plans [22]. Giżyńska et al. reported that the reduction rate was 48.7%/32.8% (MU), 13.4%/7.9% (node) and 26.5%/7.9% (beam) for prostate/lung plans [23]. It is worth to point out that different approaches in optimization algorithms were used in VOLO and our approach. We also admitted that the AD-S system was compared with MLP using clinical MLP plans. More comprehensive comparison like re-optimize the MLP plans to obtain better plans, and meanwhile re-optimize the AD-S plans using these improved plans as reference could make the study more solid.

To our knowledge, it will take about rather long time for MLP system to obtain a clinical practical plan. The optimization time may be several hours for large irregular target. We want to point out that the circular cone and MLP system are not well-suited for large irregular target. The recently introduced MLC together with the VOLO optimizer would surely shorten the optimization time and improved the quality of treatment plan. Applying the framework of this work for more widely used MLC treatment planning, either CyberKnife system or linac system, will be our next work in the future.

Recently the vendor introduced multi-leaf collimator (MLC) to the system. Although the MLC increased the flexibility for field size and improved the treatment efficiency for irregular shaped targets, the circular cones are still in use widely due to its highly conformal dose distribution [6]. Furthermore, the MLC system has not yet been widely adopted, with only about 20% systems installed globally at the current time according to the vendor. Therefore, any improvement in the circular cone based plan optimization will have a big impact in the clinical operation of these systems, which is the aim of this study.

In our previous SVDLP algorithm we developed a linear model and solved the model with Gurobi (Gurobi Optimization, Inc., Houston, TX, USA). The improvements of AD-S over SVDLP include: (1) Both beam and node reduction was integrated into the model; (2) The optimization model was free from the limitation of linearity, and the dose-volume constraints could be directly added; 3. Open-source toolkit replaced the commercial solver. For the same brain (patient 3) and liver (patient 3) cases of our previous study, the AD-S plans achieved comparable dose distribution but used fewer nodes and beams than SVDLP plans. In addition, the computational time was 627 s and 285 s for the SVDLP approach, and 84.8 s and 32. 1 s for AD-S approach, almost an order of magnitude improvement.

Our results demonstrated that the AD-S approach used fewer nodes and beams and lower MU to achieve same or better dose distribution than MLP. In other words, the treatment time was shortened without jeopardizing the treatment quality. This was because the penalty on the numbers of beams and nodes was modeled with the *lasso* and *group lasso* terms and took effect during optimization. The MLP system used an alternative method, which is to directly eliminate the beams and nodes with marginal weights after optimization, i.e., the optimization and reduction procedures were performed separately. It was plausible that the reduction procedure would deteriorate the optimization results significantly. So was the case of the round-ended PTV DVH curve (lower minimum dose) of the brain case, which was not clinically desirable, especially for SRS treatment. The VOLO optimizer has also overcome this limitation by integrating beam reduction with optimization.

The AD-S approach used the same sets of cones with MLP system, which was selected empirically by experienced physicist. How to choose the optimal cones were not covered in this study. Intuitionally, the optimal cone size is directly related with the size and geometric irregularity of the PTV. Considerable researches have been published [24–26], which showed that the quality of treatment plan would be improved even with simple empirical formula or mechanism. However, this issue has not been fully addressed, which could be explored potentially with the DL technique. The fast AD-S approach proposed makes it possible to test more cases with varying complexities and fulfill the task of big-dataset construction for further DL research.

## Conclusion

We have developed an AD-S approach for circular collimator based robotic radiotherapy treatment plan optimization. The optimization model was topologized into a simple neural network, and the automatic differentiation approach was adopted to solve the model. The *lasso* and *group lasso* regularization terms were utilized for beam and node reduction. Our investigation in brain and liver cases demonstrated that the AD-S approach could quickly achieve at least comparable treatment plans using fewer beams, nodes and lower MU.

## Supplementary Information

**Additional file 1**. The key codes of using pytorch for optimization.

## Data Availability

The datasets generated during and/or analyzed during the current study are not publicly available, but can be inquired from the authors.
