# Supplementary material for: Applying pytorch toolkit to plan optimization for circular cone based robotic radiotherapy
Source: Radiat Oncol. 2022 Apr 20;17:82. doi: 10.1186/s13014-022-02045-y (PMC9022303; doi:10.1186/s13014-022-02045-y)
Supplement: Supplementary file 1 — Additional file 1. The key codes of using pytorch for optimization. [file 13014_2022_2045_MOESM1_ESM.docx]

**Example of using pytorch for treatment planning**

We will demonstrate how to use pytorch for treatment planning with a simple case. The key codes are also presented. Suppose the case contains a single PTV, and the constraints are >2000cGy and <2300cGy. It will be easy to deduct the implementation of complicate cases with this example.

1. **Data preparation:**

The dose delivered to all PTV voxels by all beams were calculated (see eq. 2.1). The calculated data (d_ij_) is referred as *input_data.*

1. **Network construction**

The network was defined as:

*import torch.nn as nn*

*class network(nn.Module):*

*def __init__(self,n):*

*super(net_sqr, self).__init__()*

*self.weight = torch.nn.parameter.Parameter(torch.Tensor(n, 1))*

*def forward(self, x):*

*return x.matmul(self.weight)*

1. **Define loss function**

The loss function was defined as:

*def loss(y_pred):*

*cst_min= (y_pred - 2000)**2* y_pred.le(2000).float()*

*cst_max= (y_pred - 2300)**2* y_pred.gt(2300).float()*

*return cst_min+ cst_max*

le and gt are the pyrorch functions. if the value is less than 2000 or greater than 2300, it will return 1 otherwise 0.

1. **Define closure function**

The closure function was defined as:

*def closure():*

*optimizer.zero_grad()*

*y_pred = model(input_data)*

*return loss(y_pred)*

1. **Preform Iteration**:

Then the optimization was performed:

*import numpy as np*

*import torch*

*import torch.optim as optim*

*model = network(np.shape(input_data)[0])*

*input_data = Variable(torch.Tensor(opt_data.kr))*

*optimizer = optim.LBFGS()*

*for i in range(itr_time):*

*optimizer.step(closure)*

*beam_weight = (model.weight).data.numpy()*

The optimized beam weights were *beam_weight*
